# Pituitary Disorders and Osteoporosis

**DOI:** 10.1155/2015/206853

**Published:** 2015-03-19

**Authors:** Marek Bolanowski, Jowita Halupczok, Aleksandra Jawiarczyk-Przybyłowska

**Affiliations:** Department of Endocrinology, Diabetes and Isotope Therapy, Wroclaw Medical University, Pasteura 4, 50-367 Wroclaw, Poland

## Abstract

Various hormonal disorders can influence bone metabolism and cause secondary osteoporosis. The consequence of this is a significant increase of fracture risk. Among pituitary disorders such effects are observed in patients with Cushing's disease, hyperprolactinemia, acromegaly, and hypopituitarism. Severe osteoporosis is the result of the coexistence of some of these disorders and hypogonadism at the same time, which is quite often.

## 1. Introduction

Osteoporosis is defined as a loss of bone mass and strength that leads to fragility fractures [1]. Secondary osteoporosis results from a large and diverse group of medical problems, including endocrine disorders, adverse effects of medications, immobilization, disorders of the gastrointestinal or biliary tract, renal disease, and cancer. Some pituitary diseases may influence bone mineral density (BMD) and bone turnover causing osteopenia, osteoporosis, and fractures [2]. Moreover, in some cases fractures occur despite normal bone density. In our review we have focused on the association between osteoporosis and pituitary disorders such as Cushing's disease, hyperprolactinemia, acromegaly, and hypopituitarism. The mechanisms of bone effects of certain hormones involved in pituitary disorders are shown in Table 1, and their influence on bone turnover, bone density, and fracture risk is shown in Table 2.

## 2. Cushing's Disease

In current review we discuss both pituitary-dependent Cushing's syndrome (Cushing's disease) and adrenal-dependent Cushing's syndrome, since the final bone effects of both are caused by hypercorticism. Glucocorticoid-induced osteoporosis (GIO) is the most common cause of secondary osteoporosis. Hypercorticism affects bone metabolism in a variety of mechanisms [3]. The most significant influences of glucocorticoids (GCs) are their inhibitory effects on bone formation, acting directly and indirectly by regulating the synthesis of factors present in the bone microenvironment [4]. GCs reduce replication and prevent the terminal differentiation of bone forming cells and additionally promote apoptosis of osteoblasts and osteocytes by activating caspase 3. Besides decreasing the overall number of osteoblasts, GCs also exert effects on their function by inhibiting the synthesis of type I collagen, the major component of bone extracellular matrix, with a consequent decrease in bone matrix available for mineralization. Furthermore, alkaline phosphatase and osteocalcin serum levels are decreased in patients with Cushing's syndrome. Indirect actions of GCs involve decreased production of insulin-like growth factors (IGFs) and shifting transforming growth factor-*β* (TGF-*β*) binding [4, 5]. GCs enhance osteoclastogenesis by increasing the production of RANKL (receptor activator of NF-kappaB (RANK) ligand) and reducing the synthesis of osteoprotegerin in stromal and osteoblastic cells [3, 5, 6]. GCs prolong the life span of mature osteoclasts by acting directly or through the increased expression of macrophage colony-stimulating factor (M-CSF) [3]. Impaired intestinal calcium absorption and renal calcium reabsorption affect bone metabolism in patients with Cushing's syndrome as well. In addition, GCs decrease the release of growth hormone and gonadotropins which may also play a role in bone turnover [7]. The catabolic effects of GCs on muscle could contribute to the fracture risk because muscle weakness increases the incidence of falls. Furthermore myopathy and muscle weakness induce bone loss by removing the stimulating activity on the bone [5, 8].

GIO occurs in two phases, a rapid early phase in which BMD is reduced as a result of bone resorption, followed by a slower, progressive phase in which BMD declines because of impaired bone formation [3]. In patients with endogenous or exogenous hypercortisolism bone loss is more severe in trabecular bone than in cortical bone. This is explained by greater surface-to-volume ratio of trabecular bone compared with cortical bone; thus trabecular bone responds more rapidly to stimulation than cortical bone [7]. It is well known that patients with Cushing's syndrome may have lower BMD and increased osteoporotic fracture risk [6, 7, 9]. A study on 58 patients with pituitary-dependent and 21 patients with adrenal-dependent Cushing's syndrome showed low mean BMD before treatment. BMD values were lower in the lumbar spine than in the femoral neck, although the difference was not statistically significant. According to World Health Organization (WHO) criteria 82% patients were diagnosed with osteopenia. Interestingly, the prevalence of osteopenia and osteoporosis was independent of age. There was no significant difference in BMD between patients with Cushing's syndrome of different origins, neither in the whole group nor in male or female patients separately. Factors such as type of Cushing's syndrome, mean 24-hour serum level of cortisol, and duration of symptomatic glucocorticoid overproduction had no influence on* Z*-*scores*. Body mass index (BMI) and menstrual status did not show a correlation with* Z-score*. In males as well as in females, there was positive correlation between age and* Z-score* at both the lumbar spine and the femoral neck [7].

Endogenous hypercortisolism changes bone structure and causes vertebral fractures in up to 70% of patients. Factors associated with fracture risk include age at the onset, span and severity of the disease, and individual susceptibility to GCs [3]. In patients with endogenous Cushing's syndrome fractures occur most commonly at the thoracic and lumbar vertebrae, hip, ribs, and pelvis, not infrequently developing spontaneously or after low-energy trauma [10]. The prevalence of osteoporosis and atraumatic fractures is higher in adrenal Cushing's syndrome than in pituitary Cushing's syndrome [11]. In both endogenous and exogenous hypercortisolism fractures might be asymptomatic and occur in patients with normal or only slightly decreased BMD values [3, 10, 12]. It is thought that even subclinical hypercortisolism (SH) might be the cause of osteoporosis and increased risk of vertebral fractures [3, 13]. Morelli et al. studied the risk of new vertebral fractures in patients with adrenal incidentaloma (AI). BMD in subjects with and without SH was comparable; however the prevalence of vertebral fracture and spinal deformity index (SDI) were significantly higher in SH subjects than in the group of patients without SH. The presence of SH was related with worsening of SDI and occurrence of a new vertebral fracture or the progression of the grade of a preexisting vertebral deformity regardless of age, gender, BMI, years since menopause, lumbar spine BMD, and basal SDI. Patients with SH showed higher fracture incidence in spite of stable BMD throughout the observation [13]. This suggests that factors other than BMD, such as bone quality, are associated with increased risk of fracture [7, 13]. Although dual-energy X-ray absorptiometry (DXA) is considered a gold-standard measurement of BMD, it cannot be employed to assess bone microarchitecture, the most important factor in determining bone quality [10, 14]. The trabecular bone score (TBS) has been recently introduced as a method of bone quality estimation in GIO [10, 15]. TBS can be used in addition to BMD to divide patients according to their fracture risk. The patients with low BMD and low TBS will present fractures more likely than patients with low BMD and high TBS [16]. Eller-Vainicher et al. showed that, in AI patients with subclinical hypercortisolism, TBS is reduced and correlates with the number and severity of vertebral fractures and with the degree of cortisol excess [16].

The aim of medical therapy is to prevent fractures [3]. Initially, factors influencing the fracture risk should be recognized. Patients with GIO and all patients exposed to glucocorticoid excess should be treated with appropriate supplementation of vitamin D and calcium [12]. Administration of calcium (1500 mg daily) and vitamin D (800 IU daily) is recommended [3]. The management in patients with GH and sex steroids deficiencies may include replacement therapy with adequate hormones. It seems that treatment of GHD protects the skeleton from the adverse effects of glucocorticoid overtreatment in hypopituitary patients [3, 17]. A proactive preventive approach including lifestyle changes, such as tobacco cessation, reduction in alcohol consumption, exercise program, and restriction of sodium intake in the presence of hypercalciuria, should be implemented [5]. Antiosteoporotic drugs may be needed in patients in whom fractures risk could not be normalized by correction of hypercortisolism. Proposed treatment protocol in patients with endogenous hypercortisolism comprises bone active treatment for postmenopausal women or men older than 50 years with a 10-year risk for fractures of at least 20% (calculated by FRAX) or in patients older than 70 and/or with BMD < −1.5 SD and/or prevalent fractures and/or more severe hypercortisolism. Young patients (premenopausal women or men aged less than 50 years) with long-term pituitary hypercortisolism, even subclinical, not adequately corrected by surgery should be treated with antiosteoporotic drugs [12]. Bisphosphonates ought to be considered in patients with Cushing's syndrome who have a decreased BMD, regardless of age [7]. Administration of alendronate or clodronate provides faster BMD recovery in comparison with untreated patients [3]. Fracture risk (FRAX score) and BMD should be assessed few months after resolution of hypercortisolism. Based on that, clinician is able to make a decision about cessation of antiosteoporotic therapy [12].

## 3. Hyperprolactinemia

Hyperprolactinemia causes secondary hypogonadism and may have sex hormone-independent effects on bone metabolism [17, 18]. Estrogen deficiency in approximately 50% of premenopausal women with secondary amenorrhea is attributable to acquired gonadotropin-releasing hormone (GnRH) dysregulation, comprising hyperprolactinemia. The effects of estrogen deficiency on bone include an acceleration of bone turnover with higher resorption than formation [19]. In cases of functional GnRH deficiency the pattern of GnRH secretion is altered, involving reduced frequency of pulsations and decreased pulse amplitude [4, 5]. Trabecular bone is more affected than cortical. A study on hyperprolactinemic males showed low BMD at the lumbar spine in 80% of patients, whereas only 30% subjects had a reduced BMD at the femoral neck. Similar finding was reported in women [20].

Increase in bone resorption and low BMD occur in males and females with hyperprolactinemia [18, 20–23]. Authors reported that the patients with prolactinomas also presented with osteopenia or osteoporosis and the risk factors of bone loss were disease duration and hypogonadism [21]. A significant negative correlation was found between lumbar spine and femoral neck BMD values and both prolactin (PRL) levels and disease duration, which suggests that the duration of the disease is strongly correlated with the severity of bone loss [20, 22]. In addition, patients with a longer duration of hypogonadism are likely to have lower bone density [19, 22]. In men with prolactinoma the absence of detectable levels of estradiol was correlated with lower BMD in all analyzed sites. It is not clear if testosterone deficiency or estradiol deficiency is mostly responsible for bone loss in men with hypogonadism [22]. The importance of achievement of adequate peak bone mass is considered the best protection against age-related bone loss. The disturbances impeding the acquirement of peak bone mass severely influence future bone health. Patients who developed hyperprolactinemia in childhood or adolescence have more severe bone impairment than subjects who developed hyperprolactinemia in adulthood [20, 22]. The evaluation of bone markers can be useful in diagnosing an early bone turnover alteration before a change in BMD becomes apparent. In one study, in all patients, osteocalcin (OC) levels were significantly lower and urinary cross-linked N-telopeptide of type I collagen (Ntx) levels were significantly higher; however BMD values were in normal ranges in 20% of the patients [20].

Untreated prolactinomas were associated with a significant increase in fracture risk attributed to gonadotropins and sex steroids deficiency or hyperprolactinemia per se [17]. Mazziotti et al. have shown that higher prevalence of radiological vertebral fractures (VFs) occurs in women and men with PRL-secreting adenomas compared to controls [18, 24]. Males with prolactinomas who suffered fractures had lower BMD* T-score* and longer duration of the disease, independently of the effects of age, serum IGF-1 and PRL values, frequency of macroadenomas, adrenal insufficiency, hypothyroidism, diabetes insipidus, parental history of fractures, cigarette smoking, and excessive alcohol consumption [18]. In female patients prevalence of VFs was associated with the duration of the disease independently of the effects of hypopituitarism, age, BMD, serum PRL levels, and treatment with dopaminergic drugs [24]. Fractures were more frequent in patients with untreated hyperprolactinemia in comparison with patients treated with cabergoline [18, 24]. Dopamine agonist therapy restores gonadal function and increases vertebral BMD in most hyperprolactinemic women [19, 20]. History of amenorrhea may be a cause of an increased fracture risk despite resumption of menses [19]. A progressive significant increase in serum OC levels and a significant decrease in Ntx levels were noted after 6, 12, and 18 months of treatment in the 3 groups of patients. 18-month treatment with one of the dopamine agonists (bromocriptine, quinagolide, or cabergoline) normalized serum PRL and OC levels and gonadal function, although it was unable to completely restore lumbar spine and femoral neck BMD and normalize Ntx levels [20].

## 4. Acromegaly

Acromegaly is characterized by growth hormone (GH) excessive secretion and in most cases is caused by the presence of a somatotroph tumor of the pituitary gland [25, 26]. GH and its main peripheral mediator insulin-like growth factor-1 (IGF-1) stimulate proliferation, differentiation, and extracellular matrix production in osteoblastic cells. GH and IGF-1 also promote osteoclast recruitment and bone resorption activity [27–30]. In prepubertal period anabolic action of GH is responsible for longitudinal bone growth, whereas during the adolescence and early adulthood it induces skeletal maturation until the achievement of peak bone mass. In adulthood GH regulates bone turnover, thus maintaining bone mass [27]. In patients with acromegaly changes in bone size and density and arthropathy are common problems [31–33]. Acromegaly is known to be a secondary cause of osteoporosis [27].

Information about the influence of GH excess on BMD varies in the literature. In some studies BMD was reported to be increased or within the reference ranges, while in other papers it was decreased [17, 26, 34–37]. Conflicting data on bone mineral density might be related to gonadal status, different skeletal sites being measured, various techniques employed, subject's gender, age, and activity of the disease [28, 36, 38]. Hypogonadal patients with acromegaly are at higher risk of osteoporosis, particularly at sites composed predominantly of trabecular bone, which has more intimate contact with the circulation and is influenced by sex steroids to a greater extent [34, 39, 40]. Normal or increased bone mass is attributable to anabolic effect of GH, although BMD measurements may be overestimated due to structural modification of the spine [27]. Hypogonadism occurs frequently in patients with acromegaly as a consequence of PRL and GH hypersecretion or hypopituitarism due to local mass effects [25]. Impaired gonadal status including hypogonadism and hyperprolactinemia is thought to be a cause of BMD decline [28, 34]. In one study, forearm and vertebral BMD values changed in opposite directions. It seems that forearm BMD was increased because of GH and IGF-1 action and vertebral BMD was low due to hypogonadism [41]. Axial skeleton is composed of 70% trabecular bone, while appendicular skeleton of 90% cortical bone [40]. Cancellous bone is more susceptible to rapid resorption than cortical bone. Patients with acromegaly generally demonstrate increased cortical bone mass, whereas trabecular BMD is more variable [23, 35, 41, 42]. Influence of gender and disease activity on BMD has been also widely studied [36, 40, 43]. Scillitani et al. reported that the anabolic effect of GH excess on bone in acromegalic patients is gender independent, evident only at the spine in eugonadal patients regardless of disease activity and present only at femoral neck during active disease regardless of gonadal status [36].

It is well known that in patients with acromegaly bone turnover is increased [28, 29, 35]. A specific marker of bone formation, osteocalcin, is elevated in subjects with active disease [28, 29, 35, 40, 43]. Indicators of bone resorption such as urinary hydroxyproline/creatinine, urinary type I collagen cross-linked N-telopeptide, and serum C-terminal collagen type I cross-links are in higher concentrations than in controls [28, 29, 35, 40, 43]. Additionally, serum calcium and phosphate values and 24-hour urinary calcium are elevated in patients with active disease [27, 29, 34, 35, 41]. Rise in serum calcium levels may be caused by increased intestinal calcium absorption, while high serum phosphate levels may be due to GH-mediated increased intestinal and renal absorption [27].

Bone quality depends not only on bone density and bone turnover but also on collagen integrity and the macrostructure and microstructure of bone [26]. Quantified ultrasound (QUS) measurements of skeletal properties provide important information on bone strength and resistance to fractures. This method is performed at hand phalanges and at the heel in patients with acromegaly [28, 33]. High-resolution peripheral quantitative computed tomography (HR-pQCT) permits in vivo assessment of the bone microarchitecture and the volumetric BMD in the distal radius and tibia [26]. DXA is currently the most prominent clinical tool used to assess bone health. However, DXA and QUS methods are not sufficient for identifying patients at risk for fracture, due to the many possible interferences including bone deformities, osteoarthritis, joint rigidity, and soft tissue thickening [26, 27]. Patients with acromegaly have an increased risk of fractures, which might be correlated with insufficient quality of bone [26, 27, 39, 42]. The reported prevalence of VFs varies between 39% and 59% [27, 42]. In a prospective study the incidence of VFs was significantly higher in patients with active disease as compared with those who had controlled/cured acromegaly at the beginning of the study [38]. Claessen et al. showed that VFs tend to progress in the long term in 20% of patients with biochemically controlled acromegaly in the absence of osteoporosis or osteopenia. This is suggestive of an abnormal bone quality persisting after remission, possibly due to pretreatment long-term exposure to high circulating levels of GH [30]. VFs are often asymptomatic and are largely underdiagnosed. Fractures occur more frequently in men than women. They are also more severe and often multiple in men [27, 42]. Vertebral fractures may be present in patients with normal or slightly decreased BMD, which makes BMD a poor fracture predictor [27, 30, 39, 42]. In patients with acromegaly a spine X-ray should be performed, both at diagnosis and during follow-up in order to reveal possible VFs [39, 42]. The early diagnosis and the effective control of acromegaly seem to be the most important tools in reducing the risk of VFs [38].

## 5. Hypopituitarism and Growth Hormone Deficiency

Patients with isolated GH deficiency (GHD) and with multiple pituitary deficiencies usually have low BMD. However, in some cases normal BMD can be also observed. In patients with childhood onset (CO) GHD there seems to be a clear reduction in BMD, which may present the potential role of GH in the achievement of peak bone mass [44–49]. Other authors claim that there is no evidence supporting association between isolated CO GHD and increased fracture risk or low bone density [50]. Studies have shown that GHD itself is crucial for the development of osteopenia in hypopituitary patients [44, 49, 51]. A direct relation between GHD and decreased bone mass in hypopituitarism is supported by the fact that GH replacement therapy improves BMD values [51, 52]. The causes of reduced BMD in patients with hypopituitarism and untreated GHD are not fully explained. According to the literature BMD might be affected by many factors such as age at onset, gender, body mass index, gonadal status, and the severity of growth hormone deficiency [47, 49, 51]. Pituitary hormone deficiencies causing hypogonadism, hypothyroidism, or hypoadrenalism may also contribute to bone loss. The mechanisms underlying the association between either ACTH or TSH deficiency and lower BMD are not clarified and might be a consequence of either deficiencies of pituitary hormones or excessive hormone replacement [47, 53, 54]. It is important to take into consideration possible interactions between replaced hormones [53].

It has been reported that GHD and hypopituitarism are associated with increased fracture risk [17, 44, 51, 55]. Studies suggest that fracture rate is rather attributable to GHD alone than other pituitary hormone deficiencies or their replacement therapy [51]. In hypopituitary adult males with untreated GH deficiency vertebral fractures occurred more frequently in patients who received higher cumulative and current doses of cortisone than in patients who received lower doses [54]. In one nationwide study of patients suffering from GHD the risk of fracture was significantly increased in adult onset (AO) GHD females, but not in males. This was explained by a reduced percentage of properly treated females with hypogonadism compared to men [56]. Holmer et al. studied fracture incidence in GHD patients on complete hormone replacement therapy. They demonstrated that women with CO GHD had more than doubled risk for nonosteoporotic fractures. In contrast, a significantly decreased incidence of fractures was observed in AO GHD men. These findings were justified by the interaction between oral estrogen and the GH-IGF-1 axis in women and by the adequate substitution of testosterone and GH in men [44].

Recent meta-analysis revealed that administration of recombinant human growth hormone (rhGH) resulted in a significant increase in BMD in randomized/controlled studies of more than 12 months. Similar outcomes were observed in prospective studies. This beneficial effect of rhGH replacement therapy is affected by gender, age, and treatment duration. The doses used in studies varied from 0.2 to 0.96 mg/daily with higher doses prescribed to women and patients with CO GHD [57]. BMD within the first months of treatment decreased which was followed by a subsequent increase after at least one year of therapy. GH replacement promotes bone turnover by stimulating both bone formation and bone resorption, which is prominent during the first phase of GH treatment. After this period bone turnover slows down and bone mass increases [48, 57, 58]. Some studies suggest that GH replacement therapy may reduce the risk of fracture in patients with hypopituitarism and GHD [51, 59].

## 6. Conclusions

Secondary osteoporosis and fragility fractures can be a result of Cushing's syndrome, hyperprolactinemia, acromegaly, and reduced production of sex steroids (hypogonadism). Hypopituitarism might also be a cause of bone loss due to deficiencies of several pituitary hormones, especially growth hormone or excessive replacement therapy. The key is early diagnosis and effective management of the underlying disease. In addition, primary prevention of fractures and proper treatment of osteoporosis are very important, especially in patients unsuccessfully treated with basic disease.

## Figures and Tables

**Table 1:** Mechanism of bone effects of certain hormones acting in pituitary disorders.

Hormone	Physiological effect	Effect of hormone excess	Effect of hormone deficit
GH, IGF-1	Anabolic effect, influence bone size, OB stimulation, OC promotion, necessary for attaining normal PBM	Thickening of bone, ↑cortical BMD	↓PBM, ↓BMD

Prolactin	None	OC stimulation, ↓trabecular BMD	None

Cortisol	None	OB inhibition, OC stimulation, ↓trabecular BMD, ↓PBM	None

Sex steroids	Necessary for attaining normal PBM	None	OC stimulation, ↓trabecular BMD

Thyroxine	Necessary for bone growth and attaining normal PBM	↓PBM, OC stimulation	↓PBM

OB—osteoblast; OC—osteoclast; PBM—peak bone mass; BMD—bone mineral density.

**Table 2:** The influence of pituitary disorders on bone turnover, bone density and fracture risk.

Disorder	Bone turnover	Bone mineral density	Fracture risk
Acromegaly	Increase	Increase/decrease	Increase
Hyperprolactinemia	Increase	Decrease	Increase
Cushing's Disease	Decrease	Decrease	Increase
Hypogonadism	Increase	Decrease	Increase
Hypopituitarism	Decrease	Decrease/normal	Increase
GHD	Decrease	Decrease	Increase

GHD-growth hormone deficiency.
